# Developing a tool to assess technical skills in talented youth table tennis players—a multi-method approach combining professional and scientific literature and coaches’ perspectives

**DOI:** 10.1186/s40798-021-00327-5

**Published:** 2021-06-19

**Authors:** Irene R. Faber, Till Koopmann, Dirk Büsch, Jörg Schorer

**Affiliations:** 1 Institute of Sport Science, University of Oldenburg, Oldenburg, Germany; 2 International Table Tennis Federation, Lausanne, Switzerland

**Keywords:** Technique, Talent identification, Talent development, Aptitude, Child, Racquet sports, Youth sports, Sports

## Abstract

**Background:**

The assessment of technical skills as part of a multidimensional approach for talent identification and development in sports seems promising, especially in a technique-based sport like table tennis. However, current instruments mostly focus on a single element of technical skills, mainly use quantitative outcomes, and/or are not developed for talent purposes. Practice would benefit from a new instrument using a more ecologically valid approach. Thus, the purpose of this study was to identify the essential elements of technical skills in young table tennis players and to establish a first tool while using a multi-methods study design including an archive search for professional literature, a systematic search for scientific literature, as well as ten in-depth interviews with expert coaches.

**Results:**

This approach taken ensured empirical findings to be combined with knowledge and experiences from the practical field and detailed explications by high-level expert coaches. Results for the literature searches yielded 23 professional and 21 scientific articles while data saturation was reached through all ten interviews. The triangulation process resulted in two general (i.e., individuality, interconnection between elements) and five specific (i.e., bat grip, ready position, footwork/body positioning, service, stroke) elements of technical skills in young table tennis players. In addition, criteria for both flawed and excellent executions were identified for each of the five specific elements. Finally, these results were used to create an observation sheet usable for an assessment during competition.

**Conclusions:**

This study revealed the crucial elements of technical skills that should be taken into account when assessing sport-specific technical skills of youth table tennis players (8–12 years). Moreover, it provided concise descriptions of what is considered to be flawed or excellent executions of technical skills. Based on these findings, a first observation sheet, the Oldenburg observation sheet for Table Tennis Technique (O3T), was created to be used for the assessment of the current technical skill level within a competitive context at the early stage of a table tennis player’s career. Future research should focus on its measurement properties and its value within a multidimensional assessment for talent purposes.

**Supplementary Information:**

The online version contains supplementary material available at 10.1186/s40798-021-00327-5.

## Key points

- This study reveals the value of combining findings of professional literature, scientific articles, and expertise of coaches for the development of a new instrument for talent purposes in sports while considering ecological requirements.
- The multi-method approach yielded essential elements for the assessment of the technical skills level in talented youth table tennis players (8–12 years) including both the quantity and quality of technique.
- Based on these outcomes, a first observation sheet, the Oldenburg observation sheet for Table Tennis Technique (O3T), is created for the assessment of the current technical skills level useable within a competitive context.

## Introduction

Although the pursuit of excellence is recognized in many domains, it seems highly prominent in the field of sports [1]. Most national sports associations aim for the highest level with the ultimate ambition to win medals at prestigious international sports events as the Olympic Games or World Championships. Since talent identification and development have proven to be important pillars of success [2, 3], many countries have invested largely in talent programs searching, guiding, and monitoring young talented athletes [4, 5]. Moreover, numerous attempts have been made to increase the effectiveness and efficacy of these programs including the search for performance indicators that are crucial for future success [1, 6–9]. Previous studies within various sports revealed several of such indicators that can discriminate between playing levels and/or can predict future performance to a certain extent [8, 9]. However, it is proposed that a more ecological approach, which is more representative of performance demands during competition, would increase the indicator’s utility for the elite level [9].

This connects with the findings of Koopmann et al. [10] and other researchers that particularly sport-specific technical skills measures can be helpful to discriminate between playing levels and/or predict future performance due to their close relation with the highly demanding and specialized proficiencies required for elite sports performance, even during the early phase of development [11–18]. It is suggested that the level of sport-specific technical skills could serve as one of the crucial performance indicators within a multidimensional skill set [10, 13]. Additionally, it is important to realize that technical skills interact with tactical skills; the execution of the technical-tactical strategy is always dependent on both skill areas [19, 20]. This combination of skills is a crucial component in a player’s development to be able to reach the elite level in many sports [21, 22], but specifically in sports which rely highly on technical proficiency.

Table tennis is a prime example of a technique-based sport. Players aiming for the elite level need to develop outstanding technical skills: fast switching capability to adjust stroke techniques, variable, flexible and fast footwork, pronounced ability to anticipate and react, proper positioning, and balance control [23–27]. Modern elite table tennis players cannot afford technical insufficiencies. Therefore, trainers/coaches take an emphasis on this factor from the moment a youngster starts playing table tennis when aiming for the elite level. Technical skills are considered a classical constraint in early development, and the age-span of 8 to 12 years is an important window of opportunity for high potential youth players to develop their technical skills as a fundament to be able to reach the elite level [28–30]. Thus, early mistakes that hinder a player’s technical development should be prevented [25].

Despite the importance of technical skills, systematic searches showed a gap in the field of table tennis and other racquet sports for evaluating the quality of technical skills for talent identification or development purposes [8, 31]. More specifically, there are problems with the existing measurements. First, the scarce tests currently available measure, in general, only single elements of technical skills (e.g., stroke play, stroke effectiveness or footwork) in isolation whereas integration is recommended as they interplay continuously [31]. Accordingly, performance measures that better simulate the demands of the actual competition should be developed [32–34]. Second, the outcome parameters most used in racquet sports are the speed and accuracy of the ball [31, 32, 34]. These parameters do not provide information about the quality of underlying movement patterns [13] like footwork, stroke-play, or controlling ball rotation, although this quality is essential for future potential development [25]. And third, technical evaluations are generally not operationalized and conducted from the talent identification and development perspective [8], but mainly used in high performance evaluations of adult elite players. For that reason, it is difficult to evaluate/monitor a player’s development (stage) regarding his/her technical skills.

A new instrument for the assessment of technical skills in youth table tennis players (8–12 years) following an ecological valid approach and that addresses all these elements is considered highly valuable for practice. Consequently, the aim of the current study is to find the essential elements for the assessment of technical skills in young table tennis players and establish a first tool to measure these in practice based on three pillars. First, professional literature, generally overlooked in systematic reviews [8, 10, 35, 36], is taken into account to provide crucial knowledge and experiences from the practical field with specific details on technical skills and their flawed and excellent execution. Second, scientific literature is searched to create an overview of empirical findings concerning table tennis technique that support evidence-based practice. And third, in-depth interview with expert coaches having experiences in guiding and educating young table tennis players is anticipated to ensure an even more detailed explication of the essential elements of technique including information about flawed and excellent technical performance specifically applicable to the target group. All three pillars are considered essential to build the foundation of an ecological valid instrument. Accordingly, a multi-methods design was used to triangulate the findings from professional and scientific literature and with coaches’ perspectives.

## Methods

### Design

This study used a three-part multi-methods design to identify essential elements for the assessment of technical skills in young table tennis players as the basis for a first tool for practice. The first part consisted of a specific search within professional literature available in the archives of the German and Dutch national trainers’ associations. The second part included a systematic search for scientific literature following PRISMA guidelines [37]. Results of both the first and second part were subdued to both a formal and a qualitative content analysis. The third part included a qualitative design following COREQ guidelines [38] conducting semi-structured in-depth interviews with national table tennis coaches with expertise in the field of talent identification and development. All procedures were in full compliance with the Declaration of Helsinki [39] and approved by the ethical committee of the Carl von Ossietzky University Oldenburg in Germany (Reference: Drs.EK/2020/040). Triangulation of the results from all parts was conducted and based on the findings a first observation sheet as a tool to assess technical skills in youth table tennis players was created.

### Professional literature

The archives of the Dutch and German national trainers’ associations were used for the search for professional literature (i.e., non-academic articles from trade journals/specialist literature) focusing on technical skills in table tennis. The Dutch and German archives covered the Dutch journal “VISIE” and the German journals “Trainerbrief” and “Tischtennislehre,” respectively. All articles published between 2000 and April 15^th^ 2020 were accessed online (https://www.vvtt.nl/category/visie/; https://www.vdtt.de/literatursuche) and manually screened for inclusion by two investigators (TK and IF). Articles were included if they described (the assessment of) technique or technical skills in talented or elite table tennis players. Full-text articles were consulted when the titles and abstracts did not yield sufficient information to decide on inclusion.

All articles included were first subject to a formal and then to a content analysis conducted by two investigators (TK and IF). The formal analysis was conducted to summarize the articles’ formal attributes. Since the archives of the trainers’ association covered only professional literature with a narrative approach, the extraction of characteristics was limited to publication year and country for these articles. After this formal analysis, all articles identified in the archive searches were subject to a qualitative content analysis [40, 41]. Papers were read and systematically searched in detail to distil elements for the assessment of technique, the focus on certain strokes, and their directions for flawed and excellent executions of technical skills. All findings were recorded in a spreadsheet. An inductive approach was used to code the described elements (i.e., open coding).

### Scientific literature

Electronic database searches were conducted in SPORTDiscus, Web of Science, PubMed, Scopus and SURF, limited to peer-reviewed articles published between January 1^st^, 2000 and April 15^th^, 2020. The search strategy included the search terms (“table tennis”) AND (techn* OR stroke) and was not restricted to a certain study design or sample of table tennis players (e.g., level) to find all references to different assessment methods and establish a comprehensive overview (see SI file 1 as example). Also, language was not restricted during searches. Duplicates were removed and studies that were not available as full-text publications were excluded. In addition to the search strategy used in databases, experts were consulted for additional articles. Titles and abstracts retrieved from the systematic search and expert consulting were independently screened for inclusion by three investigators (TK, IF, and MK). Studies were included if they comprised an assessment of technique or technical skills in talented or elite table tennis players (e.g., talents, high-performance players, expert, or national team players) and if they were published as original studies in international peer review journal and written in English or German. Studies were excluded when they focused on the evaluation of general motor abilities and/or handled the evaluation of an instrumental approach in motion analysis. In cases where the titles and abstracts did not yield sufficient information to decide on inclusion, full-text articles were consulted.

The data synthesis of the scientific literature followed the same procedure as for the professional literature; all articles included were first subject to a formal and then to a content analysis conducted by two investigators (TK and IF). For the formal analysis, study characteristics (e.g., publication year, sample’s country, sample size, participants’ age and sex, and study design) of all articles from the systematic search were manually extracted. Additionally, technical assessments were categorized based on both the method type and method set-up as proposed by Koopmann et al. [10]. The method type distinguishes “technique-related” (e.g., coach’s evaluation or biomechanical analysis of a technical skill) and “outcome-related” (e.g., the number of hits or maximum speed) approaches. The method set-up refers to the ecological validity and task representativeness, dividing “experimental” (i.e., lab settings, often using isolated actions) and “competition” (i.e., (video) analysis of real matches) methods. After this formal analysis, all articles identified in the systematic searches were subject to a qualitative content analysis [40, 41].

### Expert interviews

#### Participants

Ten expert, highest-level coaches (four female, six male; from 30 to 76 (*M* = 55 ± 15) years of age) were selected as a convenient sample in consultation with the German Table Tennis Federation (Deutscher Tischtennis-Bund e.V., DTTB) to participate in the interviews. Coaches had between 12 and 60 years of professional coaching experience (*M* = 31 ± 14 years) in mainly German but also international table tennis. In addition, the DTTB suggested/selected only coaches who have substantial experience regarding the education and guidance of young players and were considered to be experts in the field. All coaches held at least the highest German coaching certification (A-license) and eight of the ten were still active as coaches while the remaining two were retired but still active in talent activities by the DTTB. Written informed consent was obtained prior to each interview.

#### Interview guide and interviewers

Interviews were conducted following a narrative-based approach using an interview and topic guide (SI file 2) based on prior research and the results from the literature search. This allowed for an in-depth exploration of the coach’s perspectives regarding technical skills in young table tennis players. The interview and topic guide were tested during a pilot interview conducted by IF with an expert table tennis coach from the Netherlands Table Tennis Association before it was used for the ten interviews presented in this article.

The ten interviews were conducted by TK. At the time of the interviews, he was 28 years old and his research focused on the relevance and assessment of technical skills in the context of talent identification, selection, and development. During two of the ten interviews, TK was joined by IF. At the time of the interview, she was 40 years old and as a researcher focused on talent identification and development in various sports and in table tennis particularly. She followed a comprehensive training programme regarding qualitative research including interview training. In addition, she has been a player in national competition, is a licensed table tennis coach and has been working with youth players for approximately 20 years.

#### Data collection

All interviews were conducted with one expert coach at a time in German language to allow them to express their thoughts and ideas as precisely as possible. Starting with the open task of “Please imagine the situation that you observe a table tennis match of two young players at the age of 8 to 12.”, the expert coach was asked to describe and explicate the elements that she or he focuses on when asked to assess the players’ technical skills. Based on this introduction and the coach’s replies, an interview guide was used to deepen the various elements of technical skills named by the coach. For every important element of technical skills identified during the interview, also the characteristics of different skill/performance levels (from flawed to excellent) were considered and discussed. After the coach’s replies were discussed in detail, the interviewer potentially suggested additional, previously missing elements or thoughts based on other interviews and/or the literature search. Interviews ended when the coach was satisfied and believed his or her analysis was complete and when the interviewer covered all elements identified elsewhere. The shortest interview lasted 48 min and the longest 72 min (*M* = 58 ± 8 min). Interviews were conducted and video recorded online using the telecommunication software Skype (Microsoft Corporation, Redmond, Washington, USA). In addition, the interviewer (TK) took notes and each interview was audio recorded using Apowersoft Free Audio Recorder (Apowersoft Ltd., Hong Kong, China) and these audio recordings were transcribed using Sonix software (Sonix, Inc., San Francisco, California, USA).

#### Data analysis

Data were analyzed following inductive thematic analysis [42, 43]. At first, three authors (IF, TK, JS) both read and watched all interviews in detail developing initial codes through open coding. Afterwards, these initial codes based on the interviews as well as prior research and the literature search were discussed during multiple (online) peer debriefing and researcher triangulation meetings by, to reach consensus on codes and to generate comprehensive themes [43] and to identify related codes (axial coding). Transcripts were then managed and coded using Microsoft Word and Excel (Microsoft Corporation, Redmond, Washington, USA). The first five transcripts were coded by two investigators (IF, TK) and the coded transcripts were then compared and discussed during (online) team meetings to check coder agreement. After finding agreement between coders regarding the interpretation of the interviews and the use of codes and conducting some refinement of codes and themes, the first five transcripts were recoded/updated and the remaining five transcripts were coded by one investigator only (IF or TK). Once all transcripts were coded and analyzed, all codes and themes were reviewed in team meetings until all team members were satisfied and the raw data was reassessed to ensure the expert coaches’ perspectives were reflected. When needed, transcripts were recoded and updated. In the following, results from the interviews are presented before using it in the triangulation phase.

## Results

### Professional literature

The presentation of the results follows two steps. First, the findings of the formal analysis are described for the archive search for professional literature. Second, the qualitative content analysis is presented.

Table 1 provides a summary of the professional articles found during the archive search. This search yielded 23 articles in total, 15 from the German and eight from the Dutch archives. In contrast to the scientific literature, more articles were published in the first decade (*n* = 15 from 2000 till 2009) compared to the second (*n* = 8 from 2010 till 2020). Articles made no distinction between male and female players but handled a general approach for technique. In general, the optimal preparation and execution of technical skills were described and attention was also paid to common mistakes or flawed executions.

**Table 1:** Results archive search of professional literature

Authora	Year	Technical element/focus	Stroke
German archives			
Teichert [44]	2001	Analysis of players’ footwork during the European Championships 2000	n.s.
Nottelmann [45]	2001	Analysis of pro players’ footwork during BH strokes.	BH attack; combination with other strokes
Hammer and Zhang [46]	2002	Description of an optimal footwork and specific steps.	n.s.
Hampl [47]	2003	Description of technical skills using Timo Boll as an example.	n.s.
Krämer [48]	2003	Connection between FH and BH strokes.	BH-FH
Münzl [49]	2005	Exploring the importance of footwork stating seven hypotheses.	n.s.
Geisler [50]	2005	Discussion of beginners’ technique.	n.s.
Roscher [51]	2006	Deduction of key elements of the stroke from various professional players.	BH topspin
Voigt [52]	2007	Deduction of key elements of the stroke from Joo Se Hyuk.	BH backspin/defense
Hotz [53]	2010	Thoughts and suggestions on technical skills and their development.	n.s.
Muster [54]	2010	Reply with additions to the article by Hotz (see above).	n.s.
Schott [55]	2013	Classifications of footwork types based on literature.	n.s.
Hamrik [56]	2015	Thoughts and suggestions on bat grip.	n.s.
Krey [57]	2016	Description on how to shift the centre of mass.	n.s.
Hamrik [58]	2019	Description on how to learn this stroke.	BH topspin
Dutch archives			
Bécude [59]	2000	Description of the different phases of the stroke.	FH smash
Huber^b^ [60]	2000	Bat grip	n.s.
Lijesen [61]	2005a	Description of the different phases of the stroke.	FH topspin
Lijesen [61]	2005b	Description of the different phases of the strokes.	BH counter FH smash
Schimmelpfennig^b^ [62]	2005	Description of important elements in footwork, service, receive, active and passive play, first attacks and general elements.	Serve Receive Topspin on backspin
Lijesen [63]	2007	A stepwise approach for correcting technical flaws.	n.s.
Rieken [64]	2014	Description of the different phases of the stroke.	FH topspin
Rieken [65]	2015	Description of the different phases of the stroke.	BH topspin

*FH* forehand, *BH* backhand, *n*.*s*. not specified

^a^Only the first author is mentioned

^b^Translated from original articles from German professional table tennis journals

Based on both the open and axial coding process, it was clear that both quantity and quality of technique can be distinguished and should be taken into account. Here, quantity covers the various strokes or footwork that players can use. Nevertheless, it is worth mentioning that more than half of the professional articles (*n* = 12) did not have a specific focus on a certain stroke (articles presenting only the technique of one specific stroke were excluded), but described general elements in technique sometimes specifically focusing on footwork (*n* = 4) or bat grip (*n* = 2) (Table 1). Quality in the professional literature refers to description of how services, strokes, or footwork should be performed. For this, a kind of generic and often biomechanical approach was taken using the elite model as reference with no specific hints or emphasizes for youth players or differences between male and female players [45, 52, 59, 61, 64–67]. In general, three phases were described when attention was paid to a specific stroke: the preparation phase, the execution phase, and the recovery/end phase [51, 56, 59, 64, 65].

Technical elements distilled from the professional literature were bat grip [29, 52, 56, 68], bat angle [54, 63], bat positioning [47, 52, 57, 59], body positioning relatively to the table and the upcoming ball [44, 46, 57, 59], foot positioning [59, 63], foot work [44, 46, 49, 55, 62, 63], (remaining) balance [47, 57], timing [53, 54, 65], the use of the kinematic chain [52, 59, 61, 64–66], stroke/serve execution and their connections [48, 52, 58, 63–65], ball rotation [62, 63], ball speed [63], use of variations [62, 64], adaptation toward the upcoming ball [59, 64, 65], and effectivity and efficiency of technique [64]. Excellent performance of technical skills is considered to be related to the optimal use of the kinematic chain [52, 58, 61, 64–66]. To ensure this, players need to be able to remain their balance [47, 57], use effective and efficient foot work [44, 46, 49, 62, 63], adapt their bat grip [47, 50–52, 56, 60], and have an outstanding timing [53, 65] and stroke execution [47, 52, 63–65]. The results should be visible in the controlled speed of the bat while hitting the ball, this way creating the intended velocity and rotation of the ball. Additionally, it is mentioned that players have different preferences and styles, aligning their physical characteristics with their technical-tactical strategy to have the best performance [50, 51, 53, 64, 65] and adapting their technique to create variations of the same stroke (e.g., curved topspin versus fast topspin) [50, 53, 64, 65]. In addition, professional literature provided more detailed directions for both flawed and excellent executions of technical skills which were taken into account during the triangulation process.

### Scientific literature

Again, the presentation of the results follows two steps. First, the findings of the formal analysis are described for the systematic search for scientific literature. Second, the qualitative content analysis is presented.

**Fig. 1:**
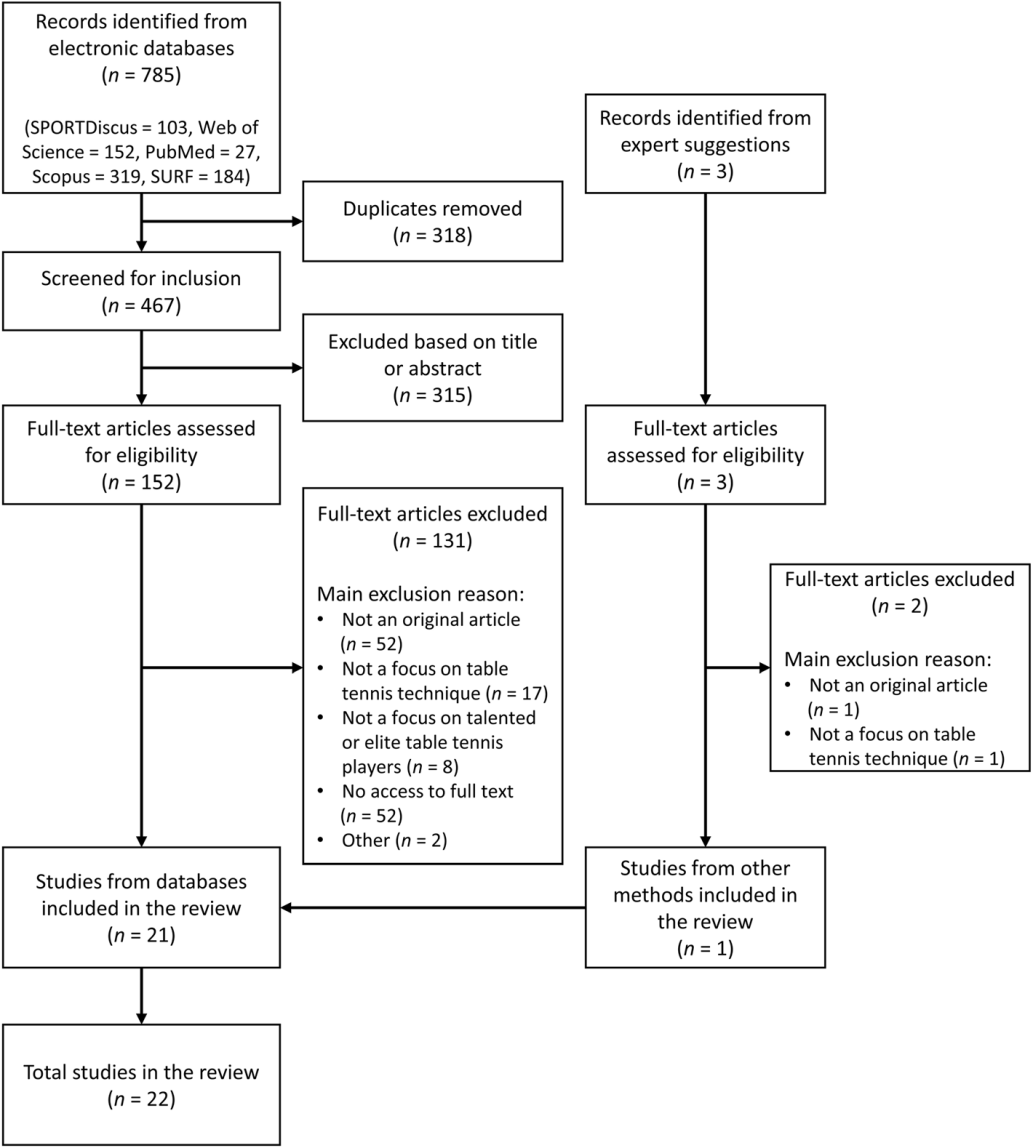
Flow-chart of systematic search for scientific literature

The systematic search in the selected databases yielded 785 studies (see flow-chart, Fig. 1). After removal of duplicates (*n* = 318) and the exclusion of studies based on title and abstract (*n* = 315), full-text articles of 152 studies were assessed regarding eligibility. From these, another 131 articles were excluded. Main reasons for exclusion were articles not being an original article in a peer-reviewed journal (*n* = 52), articles not focusing on table tennis technique (*n* = 17), or the sample not covering talented or elite players (*n* = 8). In addition, full-text articles of 52 studies could not be accessed; most of these articles were part of the China National Knowledge Infrastructure (CNKI) collection, a database not accessible to the authors. Experts suggested three additional articles of which two were excluded (Fig. 1). One article was not an original article based on empirical data and the other article did not have a focus on technical skills. Finally, 22 studies were included.

Table 2 summarizes the included results based on the systematic search within the scientific databases. Most of the included articles were published within the last decade (*n* = 19) with a remarkable increase since 2016 (*n* = 13). There was a certain global spread regarding the samples that were included. Four articles had a sample including players from different countries and/or continents. Seven of the papers included an Asian sample of players and also seven of them included European players. Only one paper covered South America and from another two papers it could not be extracted from where the sample originated. Most studies only included male players (*n* = 14) and only two included solely female players. Four studies had a mix of male and female players and of two studies, the participants’ sex was not revealed. Furthermore, it must be acknowledged that two studies included elite players with intellectual disabilities [73, 75]. All studies reporting the mean age of the sample included presented a mean age between 18 and 30 years, with an exception for the studies of Katsikadelis [34] and Mocanu [85] that included a mixed sample of youth players (mean age 13 ± 0.9 years) and female junior players (15–17 years), respectively.

**Table 2:** Results of systematic literature search for scientific articles

Author^a^	Year	Design	Sample					Method type TR/OR	Method set-up E/C	Technical element/Focus	Stroke	Findings
			Sex	*n*	Age (years ± SD)	Country	Performance level					
Marinovic [69]	2004	Comparison of four conditions (FH drive against two bounces with topspin, two bounces with sidespin, one bounce with topspin and one bounce with sidespin)	m (6), f (1)	7	27.9 ± 5.4	Brazil	Highly skilled: previous members of the Brazilian national team	OR	E	Controlling velocity of a FH drive stroke (hit, relative and absolute time, peak velocity)	FH drive	Highly skilled table tennis players need to adjust striking velocity and time (relative and absolute) to reach the peak of velocity in the forward swing phase for the task modifications.
Iino [70]	2008	Comparison of two conditions (BH topspin against topspin and backspin).	m	11	21.1 ± 4.4	Japan	Advanced table tennis players (international = 4, collegiate = 7)	TR & OR	E	Kinematic chain (contribution of joint rotations to the racket head velocity)	BH topspin	The racket upward velocity at impact was significantly higher in the backhand against backspin than against topspin, while the forward velocity was not significantly different.
Iino [71]	2009	Comparison of levels (advanced versus intermediate) and conditions (FH topspin against light and heavy backspin)	m	17	Advanced: 20.6 ± 1.2; Intermediate: 20.6 ± 1.5	Japan	Collegiate table tennis players (9 advanced and 8 intermediate)	TR & OR	E	Kinematic chain (contributions of joint rotations to the racket speed, the racket kinematics at ball impact, the time required for racket acceleration, and the maximum slope of the racket speed-time curve)	FH topspin	The advanced players showed a significantly larger contribution of lower trunk axial rotation to the racket speed at impact and a significantly larger value of the racket speed-time curve’s maximal slope, and tended to require a less time for racket acceleration than the intermediate players. The racket speed at impact was not significantly different between the two player groups. The players adjusted the racket face angle rather than the inclination of the racket path at impact to the different ball spins. The results suggest that the ability to accelerate the racket in less time in the topspin forehand against backspin balls may be an important factor that affects the performance level.
Iino [72]	2011	Comparison of levels (advanced versus intermediate) and conditions (FH topspin against light and heavy backspin)	m	17	Advanced: 20.6 ± 1.2 Intermediate: 20.6 ± 1.5	Japan	Collegiate table tennis players (9 advanced and 8 intermediate)	OR	E	Racket speed (kinetics; joint forces and torques of the racket arm and the amount of mechanical energy generated and transferred)	FH topspin	Owing to a larger shoulder internal rotation torque, the advanced players transferred mechanical energy from the trunk of the body to the upper arm at a higher rate than the intermediate players could.
Van Biesen [73]	2012	Comparison of level (players with and without intellectual disability)	m (53), f (35)	88	Player with intellectual disability: male 27 ± 8 female 28 ± 8; Control group male 24 ± 12 female 20 ± 10	Multiple	Elite players with intellectual disability (71) and a regional control group without intellectual disability (17)	TR	E	Technical proficiency	Contra, topspin, block, push, and smash	These results suggest that impaired cognitive functioning may have a direct bearing on technical proficiency in sport.
Malagoli Lanzoni [74]	2014	Comparison of styles (European versus Asian playing styles)	m	20	26.8 ± 4.7	Europe/Asia	Elite players (world's top 30 players; European 6 and Asian 14)	TR & OR	C	Bat grip, type of stroke (push, flick, topspin, block, top-counter-top, lob, smash, drive), type of footwork (one step, chasse, slide, pivot, crossover, no step), shot outcome (winner, return, error)	All	A strong association was found between strokes and footwork types, with most stroke types executed each after specific footwork types. When compared to Europeans, Asians used more frequently the most aggressive strokes and footwork types, confirming anecdotal claims on their particularly offensive playing style. Asians showed also a better serving effectiveness, often sending the ball in those areas of the table from which a counterattack is difficult to make.
Katsikadelis [34]	2014	Reliability study	m (16), f (14)	30	13.3 ± 0.9	Greek	Youth players with 3 years training experiences, training at least 4 times a week	OR	E	Hits within a certain amount of time (repetitive accuracy task).	FH and BH topspin, flip, counter-drive	From the results it is shown that the overall reliability of the studied TTSBT was high (*a* = 0.85). However, 3 tests were excluded from the testing battery due to their low reliability. In conclusion, the TTSBT is generally a reliable test battery which focuses on the evaluation of the technical skills and the table tennis performance progress of the young players.
Van Biesen [75]	2014	Comparison of simulation testing and game play	m (13), f (11)	24	25 ± 6	Multiple	Top 16 players of 2009 with intellectual disabilities	TR	E & C	Technical proficiency	Flick, FH topspin, BH topspin, contra, block, and push	Ratings of overall technical proficiency were not significantly different between Simulation Testing and Game Play conditions. There was a strong positive correlation between technical proficiency measured during Game Play vs. Simulation Testing for the variables flick, topspin forehand, and topspin backhand. No correlations were found for the variables contra, block, and push.
Suzuki [76]	2015	Comparison of levels (advanced versus intermediate) and conditions (six temporal constraint conditions)	?	14	College-age players	Japan	College-age players (7 advanced and 7 novices)	TR & OR	E	Robustness of stroke trajectories and hitting accuracy	FH and BH	Robustness to the temporal constraint was higher for the advanced players than for the novices.
Fu [77]	2016	Comparison of levels (superior versus intermediate)	m	26	?	Japan	National first (13) and second division (13) players	TR	E	Center of pressure trajectory	FH topspin loop	Compared to intermediate players, superior players showed significantly larger medio–lateral COP displacement at backward-end and significantly smaller anterior–posterior displacement at both backward and forward ends. In addition, the ratio of COP velocity between forward swing and backswing was much higher for superior subjects. Results indicated that superior players possessed better foot drive technique and ability of foot motion control during forehand loop.
Le Mansec [78]	2016	Comparison of levels (expert versus advanced versus inexperienced)	m	52	26.1 ± 8.7	France	Expert (20), advanced (14), and inexperienced (18) players	OR	E	Stroke performance: ball speed, accuracy and performance index (speed*accuracy/100)	FH topspin	Ball speed and accuracy were greater in experts than in the other groups, and both ball speed and accuracy were correlated with the level of the players.
Padulo [79]	2016	Comparison of levels (elite versus intermediate versus non-table tennis players)	m	28	Elite: 24.71 ± 2.99; Intermediate: 29.50 ± 2.26; Not playing: 28.87 ± 2.56	Italy	Elite (11), intermediate (6) and not playing (11)	OR	E	Ball speed *(in addition also reaction time and response time)*	FH and BH	In the ball speed test the elite were constantly faster compared to the intermediate group in both forehand stroke and backhand stroke. Overall, the forehand stroke was significantly faster than the backhand stroke.
Qian [80]	2016	Comparison of levels (superior versus intermediate)	m	26	Superior: 20.1 ± 0.9; intermediate: 21.2 ± 1.6	China	Professional players of university team (13 superior and 13 intermediate)	OR	E	3-D lower limb kinematics (joint angles of ankle, knee and hip, angular changing rate) and foot contact area	FH loop	Superior players showed significantly larger hip flexion and knee external rotation at backward-end and larger hip internal rotation and extension at forward-end compared to intermediate players. Foot contact areas at both events were larger for superior players. In addition, superior players showed significantly larger joints angular changing rate during forward swing at the ankle and hip. Results indicated that superior players possessed better ability of using lower limb drive in forehand loop.
Bańkosz [81]	2017	Comparison of strokes	f	12	20.0 ± 5.5	Poland	High level players (ranked TOP 16 in juniors or seniors)	OR	E	Duration of shots, distance (trajectory) of the racquet, racquet velocity	FH topspin and BH topspin	The longest racquet trajectory was related to forehand shots, shots played against a ball with backspin and winner shots. The maximum racquet velocity was precisely in the moment of impact with the ball, which is probably the most important principle in playing technique.
Zhang [82]	2017	Comparison of level (experts vs. novices)	m	20	Experts: 24.1 ± 1.6; Novices 23.1 ± 4.1	?	Professional players (10) and beginners from general university population without prior training (10)	OR	E	Stroke alignment based on the racket center motion (accuracy and phase durations)	FH stroke	Experts show higher racket resultant velocity than novices during both the forward swing and follow through phases by up to a factor of two.
Ivanek [83]	2018	Relation between technical/tactical evaluation conducted by coaches and performance.	?	48	18–36	Bosnia-Herzegovina	Top senior players	TR	E	Efficiency of play (coach evaluation (*n* = 5))	All	Results indicate that technical and tactical characteristics have a major impact on the player’s performance and are essential for table tennis success.
Malagoli Lanzoni [84]	2018	Comparison of strokes (cross-court versus long-line topspin)	m	7	22.2 ± 3.2	Italy	Competitive players of the first and second national league	TR & OR	E	Kinematic chain	FH topspin	Significantly more flexed right knee and elbow angles were measured at the moment of maximum velocity of the racket (MMV) in long-line FH topspin. In addition, significantly greater angles between the feet and the table and between the shoulders and the table at the MMV, indicate more pronounced rotation angles of the lower upper and upper-body in long-line compared to cross-court with respect to the table. A higher inclination of the racket at the MMV was found in long-line shots. The elbow flexion and the racket inclination may be associated to the direction of the shot.
Mocanu [85]	2018	Intervention study (experimental versus control group)	f	20	Junior I or II	Romania	Junior players (ranks 8^th^ to 133^rd^ place; 10 experimental group and 10 control group)	OR	E	Correctness (hit on the table)	FH and BH topspin	The comparative data obtained from the initial and final testing in the control and experimental group validated both the increase in the efficiency of topspin and re-topspin in the attack and the opportunity to use these tests to measure the progress in the technical-tactical and specific movement skills expression.
Wang [86]	2018	Comparison of level (elite vs. amateur players)	m	20	?	?	10 elite and 10 amateur players	TR	E	Lower limb kinematics and muscle activity	BH topspin loop against backspin	The present study revealed that elite players could complete this technical motion more economically than amateur players, meanwhile, elite players were more efficient in muscle usage and showed better balance ability. Elite players prepare better for the stroke, which can lead to quicker shots, and shorter time of reaching the location of the swing with better adjustment of the swing action.
Yu [87]	2019	Comparison of level (professional versus beginners)	m	18	professionals: 23.5 ± 1.24; beginners: 22.7 ± 1.62	China	Professional players (9) and beginners (10)	TR	E	Biomechanical characteristics (kinematics and kinetics)	Footwork: chasse step	The results of the present study demonstrated that professional players possessed greater foot drive technique.
Sung [88]	2019	Multiple case-study (comparison of four top players)	m	4	Adults	China/ Germany	Top players	OR	C	Score rate, usage rate and comprehensive technical score rate	All	Technical skills of these four top players were versatile. Their styles of play were unique; all of them received excellent in the evaluation of three-staged techniques. Serve, attack after serve using forehand stroke, attack after receive using backhand stroke, continuous forehand stroke attack, and continuous backhand stroke attack were the main technical skills which could be used to predict the result of the game for winning 2017 World Table Tennis Championships in men’s single event.
Bańkosz [89]	2020	Comparison of strokes (topspin after topspin versus topspin after backspin; intra- and interindividual variability)	m	7	23 ± 2	Poland	Top 10 national players	TR & OR	E	Kinematic parameters (angles and acceleration of the hand)	FH topspin	The difference in acceleration at the moment of contact between the two types of the topspin forehand was significant, but the variability of the acceleration values was small. Large variability in the angular parameters was found, and this result was considered a manifestation of different coordination patterns in the stroke movements. It is possible that even though the players used different methods of performing the movement, they obtained similar values for some parameters (e.g., acceleration), which should be taken into account by coaches. There were small differences in many parameters within individual players, which can indicate that a player performs tasks in a similar way each time. However, there was high variability in some angular parameters, indicating that the repetitions of particular strokes were not performed in an identical way.

*TR* technique-related, *OR* outcome-related, *E* experimental, *C* competition, *FH* forehand, *BH* backhand, ? unknown

^a^Only the first author is mentioned

Regarding the method type of the technical skills assessment, 10 out of the 21 studies revealed an “outcome-related” approach while six studies used a “technique-related” approach and another six studies used a mixed approach providing both “outcome-related” and “technique-related” results. As presented in Table 2, examples of “outcome-related” measures were ball speed, racket (peak) velocity, number of shots per time-interval, hitting accuracy/ball placement, speed-placement indexes, and scoring rates. Mostly three-dimensional kinematic analyses were used to produce “technique-related” outcomes such as kinematic and kinetic parameters, timing/phase durations, and the robustness of stroke trajectories. Only three studies used an observation sheet to collect “technique-related” outcomes [73, 75, 83]. In their two studies, Van Biesen et al. [73, 75] videotaped athletes during a test battery (and competition) and had experts rate their technical proficiency based on a five-element observation sheet (ratings from 1 to 10) covering ready position, point of contact, footwork, kinematic chain, and bat movement. They combined the technical and tactical characteristics as they had experts rate (ratings of 1 to 5) players based on an eight-element observation sheet including preparation phase, attack phase, defence phase, and aspects of moving. Analyzing the studies’ method set-up, 19 articles reported on an “experimental” method set-up, whereas only two studies used a “competition” set-up. Only Van Biesen et al. [75] had a mix of both as they combined an “experimental” with a “competition” method set-up.

Again, it was obvious that both quantity and quality of technique can be distinguished and should be taken into account. Furthermore, it is worth mentioning that nine scientific articles focused completely on the forehand topspin/drive and another ten included multiple strokes (Table 2). Only two scientific papers focused solely on the backhand topspin [70, 86]. Moreover, there was one article dedicated exclusively to footwork [87]. The scientific articles provided no new directions for the essential elements of technical skills and the flawed and excellent executions of technical skills, but reinforced the findings of the professional literature based on objective measurements. The scientific literature reveals that highly skilled/advanced players outperform less skilled players regarding their technical skills. They are better at using beneficial biomechanics (e.g., using kinematic chains effectively through, e.g., foot drive techniques, trunk rotations, shoulder internal rotation) [71, 72, 77, 80, 87] in their favor to create higher bat velocities and better economics compared to their less skilled counterpart [78, 79, 82, 86]. Moreover, they have greater foot motion control and a better balance ability contributing to the higher robustness to temporal constraints when compared to less skilled players [76]. This all seems to help them to have quicker shots, better movement adjustments, and better accuracy with less errors [78]. Additionally, the results show that although the foundation of technique is similar, players differ regarding their specific technique; even at the highest level, players have different preferences and styles [74, 88]. Also, adaptation of technique to create variations of the same stroke was confirmed with objective measurements [69, 70, 81, 84, 89].

### Expert interviews

Through all ten interviews, data saturation was reached. In the following, prevalent themes from the interviews are presented. First, general elements influencing technical skills in table tennis are depicted. Then, five elements of technical skills identified as most crucial by all expert coaches are presented: bat grip, ready position, footwork/body positioning, service, and stroke. It is important to mention that all coaches repeatedly emphasized their holistic and multidimensional concept of both talent and performance in table tennis. That is, all elements and factors are interconnected, influence each other, and should not be viewed as isolated areas.

#### General elements of technical skills

When discussing technical skills in young table tennis players, many coaches emphasized the need for some individuality regarding technical skills. Coaches should use a tailor-made instead of an “one-size-fits-all” approach taking into account differences in, for example, body height, body weight, or playing style:

[…] it should never be like “That’s the rule, that’s how it must be done”, because with that you maybe help one, but at the same time you kill ten. (Coach 10)

However, the question of when to start with working on an individual technique after previously teaching the basic table tennis technique is debatable and again individual:

The technique must be adjusted based on the athlete. There is a basic technique, but at some point, depending on whether she/he has been taught the basic technique well, this can maybe be at age twelve or for another athlete at age ten or for another one at age 13, 14, this should merge into a certain individuality. (Coach 05)

In conclusion, coaches agreed that there is a certain uniformity regarding technical skills most relevant for all players in the age group of 8 to 12 years and that these should be evaluated allowing for some individual adjustments depending on the athlete (at a later stage).

Furthermore, coaches emphasized the interconnection between all elements of technical skills. All five elements depend on and influence each other. For example, a functionally tensed and active ready position allows the footwork and body positioning to be the basis for an excellent stroke quality. In addition, a flawed bat grip would hinder a player in executing excellent services and strokes. These examples show that all five elements of technical skills should be considered as a combination of parameters that all need to be acknowledged in both talent identification and development.

#### Five specific elements of technical skills

##### Bat grip

Coaches stated that the bat grip is a highly crucial element of technical skills that should be considered from the very beginning of a table tennis career. Here, a neutral bat grip benefiting both forehand and backhand strokes in equal measure appears most promising:

[…] I would be glad if every coach that takes a kid and puts a bat in its hands would ensure that it is a neutral bat grip. That is the alpha and omega for me and in my view should be that way until 13 or 14 years of age before we talk about individual playing styles and preferred strokes […](Coach 01)

A neutral (shake-hand) grip refers to a grip in which players hold the bat like they are shaking hands with no tendency for neither “forehand” nor “’backhand” strokes. The thumb is positioned at the forehand side and the index finger on the backhand side. The other fingers loosely cover the bat’s handle. This grip should not influence the wrist movements in any form and allows a player to conduct all stroke techniques. Players placing their thumb and/or fingers on the blade or gripping the bat either too high or too low are seen as flawed:

[…] very bad would be of course fingers on the blade or a far too high bat grip as it stiffens up the wrist or a far too low bat grip as one has no stability. (Coach 08)

However, coaches state that young players frequently tend to use a “forehand” or “backhand” grip having benefits for attacking strokes of forehand and backhand, respectively, but providing negative consequences for other strokes. A fixed “forehand” or “backhand” grip is seen as a technical flaw. As a somewhat intermediate level concerning bat grip, coaches specified those players playing with one particular bat grip (forehand or backhand) instead of a neutral one, but at least using its advantages:

What I am interested in when the kids are coming from the street, then I want to see that they at least play to the advantages of the bad bat grip that they accustomed themselves to. (Coach 01)

While coaches stated that it should not be expected in the discussed age group, players slightly adjusting their bat grip to every individual stroke already at this young age to create an optimal use of the benefits are considered excellent.

##### Ready position

The second important element of technical skills is the actions and motions before the first stroke and in between two strokes. Generally, coaches like to see players that are “always active and alive” (Coach 01) as this activity prepares the next stroke and allows for a quicker and better reaction. Coaches emphasize that there is no other task to do in between two strokes than getting into the ready position:

I hit the ball and come back, right? The ball is hit and then I do not have any other task than moving my feet away from the table and toward the next ball. (Coach 07)

In this context, Coach 03 referred to the “golden triangle” that is formed by the trunk and the arms and which should be used as an orientation where to hit the ball:

In front of the body. In my view, in front of the body and high, over the table. High, always high, because then one can react very quickly. In the moment the bat is low, under the table, this of course leads to a longer reaction time. (Coach 04)

In addition to the bat being held in this position, players should lean forward slightly with their knees and hips bend so that their center of gravity is in front of their body. That is, their bodyweight is shifted toward their forefeet to allow for quicker movements and increased agility:

[…] perfect is when you are always working on your forefeet, no matter how you move in that moment. Without lifting your heels up artificially, but because you are having a good balance and always have your center of gravity in front. […] This would be really perfect, because then you can move in space the quickest. (Coach 01)

##### Footwork/body positioning

As mentioned above, according to coaches, there is no other task in between two strokes than to prepare the receipt of the upcoming ball and the next stroke. A crucial part of this preparation between two strokes is footwork and body positioning. Here, Coach 09 quoted a former president of the Chinese Olympic Committee stating that table tennis was “70% footwork, 30% handwork,” this way further emphasizing the crucial role of footwork. This is also supported by other coaches that see footwork and body positioning as the very basis for table tennis performance:

[…] footwork in my view is the alpha and the omega, because everything else depends on it and because it can lead to subsequent faults. If I am standing parallel, I am definitely hitting the ball too late, then I cannot have a good technique. (Coach 03)

Accordingly, highly flawed footwork and body positioning is seen as a combination of poor feet alignment, poor balance, and poor positioning behind the table in both dimensions. Here, coaches emphasized the importance of not only lateral movements behind the table, but also movements in the anterior/posterior dimension:

I pay attention to the players’ focus on the ball. The player should not move based on specific positions she/he has learned but should move and position herself/himself in the space behind the table according to the upcoming ball, the flight curve of the upcoming ball. Yea, we have players that are moving perfectly from left to right in the standardized stroke combinations, but they are not capable of working three centimeters in the depth of the room. Yea, because they simply do not perceive the depth or were not taught to also observe and work in the depth. And this depth is a highly deciding parameter in table tennis that is also tough to train. (Coach 01)

A good distance to the table is very important. Especially in kids, in my view they are often standing way to close to the table, because they have to take a step forward and then stay there instead of moving away from the table. (Coach 08)

##### Service

Most coaches see the service as an integral part of table tennis that should be taught and trained from the very beginning including its variations:

In the beginning the kids learn standard counter services and then we of course try to make them learn the variations of services with backspin, topspin, sidespin […] So, for me, the correct execution of the service is important from the very beginning. (Coach 04)

An 8- to 12-year-old first of all has to get the feeling of what a service is in the first place. What is backspin? What is topspin? What is sidespin? What is kick? Those are the things they have to learn first. […] Those are the things they have to learn from the very beginning onwards. (Coach 05)

However, for some coaches, the service often is considered somewhat separated from the match, especially in regard to technical skills. Accordingly, some coaches see the service quality as rather additional or extra for the specific age group, but still recognize its relevance and importance:

Of course, if one already has many variations, that is awesome, but for me that would not be an exclusion criterion [in this age group], if there is not much there yet. (Coach 08)

The quotes above show the coaches’ strong belief in service variations. Here, the speed, placement, rhythm, and/or rotation of the service or ball are the elements that can and should be varied. In this regard, two factors are important: the quantity of technical skills (number of service variations) as well as the quality of technical skills (how well are these services executed).

##### Stroke

The final element of technical skills mentioned by the coaches is the stroke including all its forms and variations. In this context, coaches strongly emphasized that every stroke is a movement not just with the arm and bat, but with the whole body:

Static and only the arm does the work, that would be very poor, and very good would be when it happens in the whole chain, when from the legs to the hips, shoulders, trunk muscles, everything is engaged in the stroke. (Coach 08)

This whole-body movement involving the whole kinematic chain was a theme often noted by the coaches as the basis of the stroke that is related to other elements (e.g., footwork/body positioning). The concept of the kinematic chain describes the idea that a motion impulse is created in the legs and then transferred bottom-up through the body segments until it is transmitted to the bat and from there to the ball:

[…] I am observing the hit. When are they hitting the ball, in which phase? And to have a very good hit of the ball, that is related to the positioning relative to the ball. How is the footwork? How is the feet alignment? Hip rotation, forearm, wrist action. Yes, that is what I look for. Basically the whole kinematic chain. (Coach 03)

That is, the motion impulse created by the legs is crucial to execute the stroke cleanly. But the whole body is crucial. I am cautioning against saying the stroke is all arm or I am correcting only the arm. I always have to correct the whole body. (Coach 05)

The quotes above also show the important element of timing and location of hitting the ball. Here, referring back to the “golden triangle” already included in the ready position, according to the coaches, players should always adapt their movements relative to the upcoming ball and then hit it in front of their body.

Similar to the service, stroke variations are depending on the four factors of speed, placement, rhythm, and rotation. Here, coaches want to see players that are using different variations of these factors depending on different match situations asking for different technical and tactical solutions:

Placement is really important. Yea, when I place the ball very widely or especially along the edges, no matter if during a service, a return, a block, a spin, then I always have a big advantage if the placement is well done. And then I can vary the speed, not just increasing the speed, but also decreasing the speed. That is, always playing variable but still powerful. But what is ‘powerful’? Powerful is speed, okay, but putting pressure on the opponent is also length variation, width variation, spin variation, so really the whole package. (Coach 06)

That is, by varying their strokes, players can reach the goal of putting pressure on the opponent and forcing them toward mistakes. However, it is not enough to be able to produce a high variation of strokes; it is also important that all these strokes are automated highly reproducible and executed on a high technical level. That is, similar to services, to be evaluated as having high-level technical skills players must be able to use many stroke variations during a match (quantity of technical skills) with high level technical execution (quality of technical skills).

### Triangulation

**Fig. 2:**
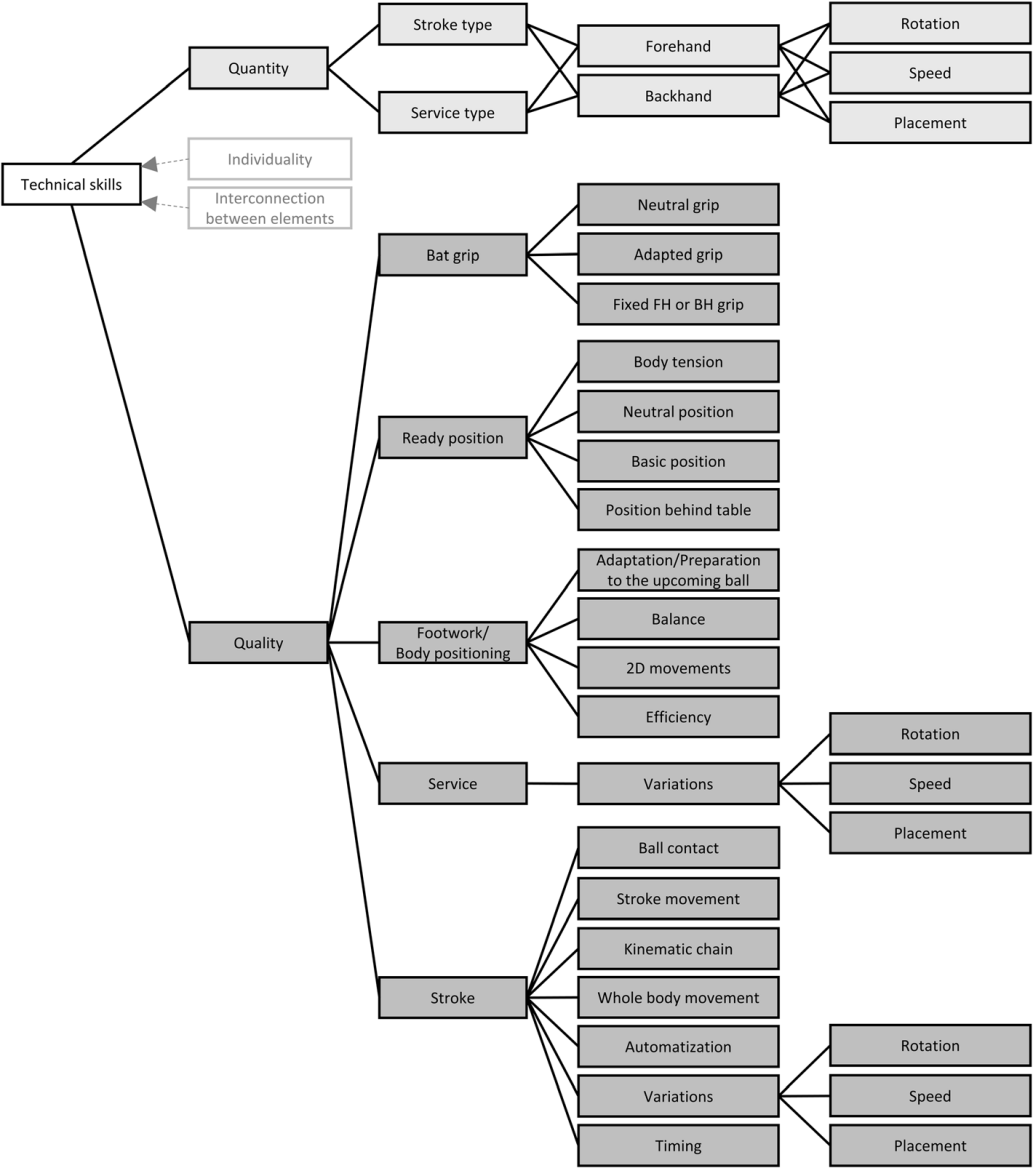
Code-tree based on triangulation

Finally, after completing both the literature searches and the interviews, all elements and findings regarding technical skills in young talented table tennis players of all parts were combined during multiple peer debriefing and researcher triangulation meetings of the research group and two expert coaches. During these meetings, all elements were discussed in every detail organizing the findings using a code tree (see Fig. 2).

**Fig. 3:**
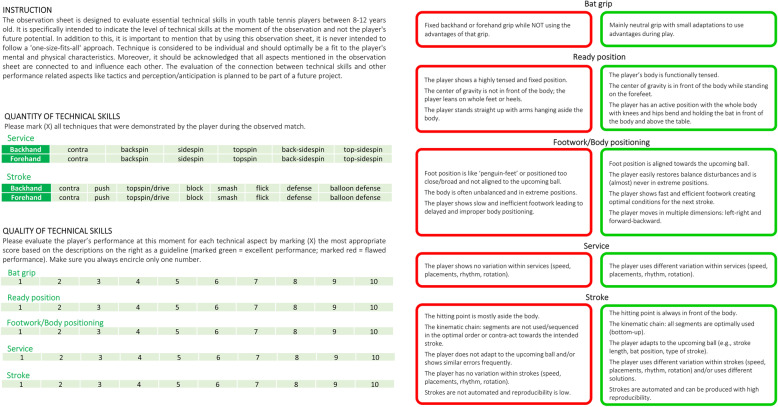
Oldenburg observation sheet for Table Tennis Technique (O3T)

This code tree includes all elements of technical skills and respective sub-categories as they were identified in both the literature and the interviews. In the end, the triangulation process resulted in a first concept of the observation sheet including a guideline on how to use it. As part of a member check, all expert coaches interviewed were invited to give feedback on this first concept to ensure the fit between the coaches’ perspectives and the researchers’ representation of these. Also, the included literature was checked again to ensure the best descriptions of flawed and excellent executions of technical skills. Feedback from the coaches and the literature findings were then used to revise the observation sheet and to create a preliminary version, the Oldenburg observation sheet for Table Tennis Technique (O3T), ready to be investigated on its measurement properties (see Fig. 3).

The O3T starts with an instructional part emphasizing three important points: (1) the observation sheet focuses specifically on the current moment instead of a longitudinal potential analysis; (2) it respects the importance of individuality in technical skills/techniques, although this importance appears to be limited in this young age group; (3) and it acknowledges both the connection between different elements of technical skills and the connection between technical skills and miscellaneous elements of table tennis performance (e.g., tactical skills or perception/anticipation). For the essential assessment part, the observation sheet is separated into two sections: the quantity of technical skills and the quality of technical skills. First, the quantity of both services and strokes covers all the different variations (i.e., forehand (FH) or backhand (BH), various spins) that a player shows during a match. Coaches mark a certain service/stroke when presented by the player. Second, the quality of technical skills refers to the observer’s ratings (from 1 (lowest/worst value) to 10 (highest/best value)) of the five specific elements of technical skills in table tennis using the criteria for flawed (red boxes) and excellent (green boxes) technical skills (see Fig. 3, right). These criteria were identified in both the literature and the interviews and represent the key technical elements and features of each technical element. The selection of the rating range of 1 to 10 was based on earlier observation tools using similar elements [73, 75].

## Discussion

The aim of this study was to find the crucial elements of the assessment of technical skills in youth table tennis players (8–12 years) and establish a tool to measure these in practice. This new instrument follows an ecologically valid approach [9] and addresses the limitations of existing instruments (i.e., focus on single elements of technical skills, “outcome-related” method type (e.g., speed and accuracy) and an operationalization for the elite adult level) [8, 31, 32]. A multi-method design was used to combine findings from professional and scientific literature with the coaches’ expertise. Two general (i.e., individuality and interconnection between elements) and five specific (i.e., bat grip, ready position, footwork/body positioning, service, and stroke) elements were distilled based on the results of the systematic search, the archive search, and the expert in-depth interviews. Moreover, concise description of flawed and excellent executions of technical skills was derived. Based on these findings, a first observation sheet for technical skills in youth table tennis players (8–12 years), the O3T, was created while paying attention to the ecological requirements. Consequently, the observation sheet is developed for the assessment of technical skills during a competitive setting. Furthermore, both quantity and quality of technique are included and all elements considered crucial at specifically the first development stage of a young table tennis player (< 12 years) are taken into account. The interviewed coaches explicated that no (large) differences regarding table tennis technique are expected between sexes at this stage, which is reinforced by literature focusing on the effect of growth and maturation on athletic performance [90]. Consequently, the observation sheet is expected to be suitable for the assessment of both girls and boys.

The multi-method approach applied in this study provided the opportunity to combine practice-based knowledge with the empirical findings published in scientific literature while each part delivered its unique contribution filling in the gaps of the other parts. Detailed descriptions of table tennis technique based on practical experiences were provided by the professional literature covering essential elements described from a general, mostly biomechanical perspective. The scientific literature reinforced these findings using objective measurements for specific elements of technique. Finally, the expert interviews added better framework and more details specifically useful for the target group of young players. The triangulation revealed that the findings of all three parts were in line with and complementary to each other; there was a strong consistency between the distilled elements of technique in table tennis and an uniformity and complementarity regarding the descriptions of flawed and excellent executions.

Moreover, a consistency/uniformity within the three parts, especially within the professional literature and the expert interviews, was found. Most professional articles presented similar descriptions of the essential technical elements and the way they should be performed. Also, the experts interviewed appeared to have a high agreement on these elements and their executions. That is, all coaches (10/10) mentioned the elements ready position, footwork/body positioning, and stroke by themselves. For bat grip, two of the ten coaches mentioned its importance by themselves while the other eight did so after the interviewer suggested discussing this element. Similarly, two coaches emphasized the importance of service themselves while seven did so after suggestion; during one interview service were not discussed. After determining each element’s importance, all coaches showed almost perfect agreement regarding the flawed and excellent executions for each element. For example, all coaches (10/10) mentioned the neutral bat grip, the active ready position, the footwork in both dimensions, the variations in services, and the use of the kinematic chain as excellent executions for the specific element. Correspondingly, similar remarks for flawed executions were given by all coaches (10/10). The very high consent between all coaches may be to some degree based on a certain philosophy or view of technical skills held within German table tennis and its association. Here, coach education plays a crucial role in establishing a certain common model and idea of technical elements in (young) players. Our results suggest that the DTTB is successfully establishing this common model within its system and future research could investigate important factors of successful coach education.

Comparing our new instrument with the observation sheets to assess players’ technical skills presented in three of the included scientific articles [73, 75, 83], one can find both similarities and differences. While Van Biesen et al. [73, 75] used similar elements (i.e., ready position, point of contact, footwork, kinematic chain, bat movement) in a different combination with less detailed criteria that were evaluated on a scale of 1 to 10, the elements used by Ivanek et al. [83] follow a distinctly different structure as they focus on different phases during a match and non-technical factors such as tactical skills and “confidence” that were rated on a 1 to 5 scale. While the observation sheet by Van Biesen et al. [73, 75] was also used for the assessment during test batteries, both the instrument by Van Biesen et al. [73, 75] and Ivanek et al. [83] are capable of assessing technical skills in an more ecologically valid setting like competition. The greatest addition with our new instrument is the detailed description of criteria for flawed and excellent executions of technical skills facilitating the evaluation process in talented youth players.

This new instrument is expected to be useful as part of a multidimensional assessment for both talent identification and development during the early phase of a player’s career (< 12 years) [10, 13]. A multidimensional assessment is suggested to reflect a player’s profile in the best way [91, 92]. Although young players with excellent technical skills probably have better chances to become successful in the future compared to less skilled players [11–18], the current observation sheet was specifically developed to measure a player’s technical skills at a certain point in time. Trainers are therefore instructed to score the technical skill level based on what they see right now and not what they expect in future. The assessment based on current performance is suggested to be more reliable compared to the prediction of future performance based on a more subjective and intuitive coach’s eye [93–95]. It provides insights in the technical strengths and weaknesses of a player useful to build up a player’s profile. Longitudinal studies are needed to reveal the observation sheet’s predictive value preferably used within a multidimensional approach [8, 9, 96]. Moreover, multidimensional characteristics among others including information about the exposure to training (e.g., quantity, quality, and education strategy/model) should be considered for a better interpretation of a players’ profile and decision-making by trainers/coaches. Thus, talent selection decisions based on a single use of the observation sheet are not recommended.

Furthermore, measurement properties (e.g., reliability, validity, and feasibility) and the exact scoring system of the observation sheet need further evaluation [97, 98]. It needs to be checked whether both the quantity and quality of technique can be observed and rated in a reliable way within a competitive setting. Previous research revealed some good prospects for the reliability of repetitive accuracy tasks [34] and distinguishing between different strokes and footwork while using (slow-motion) video-observation of elite players [99]. Nevertheless, reliability should be checked in the context where it is used to make fair conclusions [98]. For this, it is interesting to consider both the intra- and interrater reliability and also the reliability of a player’s scores when competing against different opponents. The sensitivity to changes over time and the correlation with other measures like motor performance or competition rating scores would be valuable as part of the validity analyses. Perhaps also a total score and the weight of different elements toward such a score should be considered. This further evaluation is recommended as a next step before the observation sheet’s implementation in practice.

Three limitations of this study need to be acknowledged. First, the search for professional literature was, without any intentions of discrimination or qualification, limited to the archives of two European table tennis trainers’ associations due to the accessibility and language constraints of the research group. Professional literature is generally written in the journal’s native language and not indexed in the commonly used (scientific) databases, which makes it hard to be accessed by a broad international audience. However, a broader international search might yield new insights. Second, it should be mentioned that during the systematic search for scientific literature, no full text could be accessed from 52 studies. Most of these articles were part of the China National Knowledge Infrastructure (CNKI) collection, a database providing full-text articles from more than 2000 Chinese journals. It is likely that some peer-reviewed articles handling technical skills were missed. However, from the abstracts, it seems that no young table tennis players were investigated in these articles. Third, all ten coaches that were interviewed were educated mainly in the German table tennis system and have worked (together) for the German’s national table tennis association for many years. Interviewing experts from other countries or parts of the world would potentially lead to other insights and elements regarding technical skills in young players. However, given their highest-level performance, their international coaching experience and their international networks within global table tennis, the coaches interviewed in this study probably gave highly valuable and trustworthy insights that, in combination with the literature analyses, deepen our understanding of technical skills in youth table tennis.

## Conclusion

In conclusion, the results of this study revealed the crucial elements of technical skills that should be taken into account when assessing sport-specific technical skills of youth table tennis players (8–12 years). Moreover, it provided concise descriptions of what is considered to be flawed or excellent executions of technical skills. Based on these findings, a first observation sheet, the O3T, was created to be used for the assessment of the current technical skill level within a competitive context. This new instrument is specifically designed to assess most essential elements of technical skills during the early stage of a table tennis player’s career and measure both quantity and quality in a more ecologically valid approach. Future studies should focus on the measurement properties and its added value for a multidimensional assessment for talent identification and development. This study reveals the high value of combining findings of professional literature, scientific studies, and expertise of coaches for the development of a new instrument for talent purposes in the area of sport while taking into account ecological requirements. It emphasizes the importance of connecting science and practice and presents a way how scientist and practitioners can address this. Coaches and scientists working in the field of talent, specifically those in table tennis, will benefit from the findings and the approach taken to create this new instrument for the assessment of technical skills in a highly technical-based sport combining a both “technique-related” and “outcome-related” method type during a “competition” method set-up.

## Supplementary Information

Additional file 1: **Supplementary Information 1.** Systematic search strategy for database PubMed (see Part 2 in article). **Supplementary Information 2.** Interview guide used for expert interviews (see Part 3 in article).

## Data Availability

The datasets generated and/or analyzed during the current literature review are available from the corresponding author on reasonable request. The data of the expert interviews cannot be made publicly available for ethical and legal reasons; the public availability would compromise confidentiality and/or participant privacy. The data contains potentially identifying information.

## Abbreviations

PRISMA: Preferred Reporting Items for Systematic Reviews and Meta-Analyses
COREQ: COnsolidated criteria for REporting Qualitative research
DTTB: Deutscher Tischtennis-Bund e.V.
