# Supplementary material for: Developing a tool to assess technical skills in talented youth table tennis players—a multi-method approach combining professional and scientific literature and coaches’ perspectives
Source: Sports Med Open. 2021 Jun 19;7:42. doi: 10.1186/s40798-021-00327-5 (PMC8214643; doi:10.1186/s40798-021-00327-5)
Supplement: Supplementary file 1 — Supplementary Information 1. Systematic search strategy for database PubMed (see Part 2 in article). Supplementary Information 2. Interview guide used for expert interviews (see Part 3 in article). [file 40798_2021_327_MOESM1_ESM.docx]

**Developing a tool to assess technical skills in talented youth table tennis players - a multi-method approach combining professional and scientific literature and coaches’ perspectives**

Irene R. Faber^1,2†‡^, Till Koopmann^1†^, Dirk Büsch^1^, Jörg Schorer^1^

^1^Institute of Sport Science, University of Oldenburg, Oldenburg, Germany

^2^International Table Tennis Federation, Lausanne, Switzerland

ORCIDs:

Irene Faber [https://orcid.org/0000-0002-4994-0453](http://orcid.org/0000-0002-4994-0453)

Till Koopmann <https://orcid.org/0000-0002-3985-4084>

Dirk Büsch <https://orcid.org/0000-0003-2632-5308>

Jörg Schorer <https://orcid.org/0000-0002-4888-7048>

^†^These authors share first authorship.

^‡^Corresponding author:

Irene R. Faber, PhD

Institute of Sport Science, University of Oldenburg,

Ammerländer Heerstraße 114-118

26129 Oldenburg

phone +31 6 200 30 686

e-mail: [irene.faber@uol.de](mailto:irene.faber@uol.de)

**Supplementary Materials**

**Supplementary Information 1**

Systematic search strategy for database PubMed (see Part 2 in article)

Database: PubMed (<https://pubmed.ncbi.nlm.nih.gov/>)

Search mode: PubMed Advanced Search Builder (<https://pubmed.ncbi.nlm.nih.gov/advanced/>)

Search terms: All Fields: ‘table tennis’ AND (techni* OR stroke)

Limits: Publication date: Start date; Custom Range: 2000 01 01 (YYYY MM DD)

Article type: Journal Article

Language: Dutch, English, German

Species: Humans

**Supplementary Information 2**

Interview guide used for expert interviews (see Part 3 in article)

| **Interview part** | **Description** |
| --- | --- |
| Introduction/Thanks for participating | “Thank you for participating in this project. The DTTB and we as a research group appreciate your effort concerning this study and hope you will enjoy the interview.” |
| Information letter/ Informed consent | “Before we can start with the interview, we need to make sure that you are well informed about the purpose of the study, the approach of the interview and how we deal with the data.  For this we send you an information letter that includes all these issues. Did you receive this information?  Do you have any questions before we start?” |
| Informed consent | Is a signed informed consent provided? |
| Start recording | “From this point in time we start recording the interview. Is that okay? Moreover, it can be that I make some short notes to better structure the interview.” |
| The aim of the project and interviews | “Just to start with, I will repeat the aim of the project and the interview shortly. The aim of the project is, finally, to have developed an instrument that can be used to assess technical skills in young table tennis players between 8-12 years. This instrument will be an observation tool that should be used during the scouting of young players during tournaments or events alike.  The aim of the interview is to find out what should be covered by the observation tool and how. That means we are looking for the various aspects that should be included that combined provide a player’s technical profile. Moreover, we want to be able to score these aspects on their quality. This means we need to know when an aspect of technique is scored well and when not.” |
| Aspects of technique | “If you would imagine yourself watching a youth player between the age of 8 to 12 years during a match and you want to make an evaluation of his/her technical skill level, what are the things you would to consider?”  (e.g., bat grip – footwork – balance – ready/starting position – timing of hitting the ball (relative distance to the body, table/curve) – strokes – combination of strokes – rotation – speed – accuracy – variability – service – FH topspin, …) |
| Quality of the aspects | “If you consider …; what is the perfect performance? And what would be the worst or flawed performance? Can you give examples of the ‘in-betweens’?” |
| Additions | “We talked about a lot of issues. Is there something that you would like to add? Did we forget about anything?” |
| End | “We are now at the end of the interview. I will make sure that the content will be analyzed together with the other interview. When ready, we will send you an overall summary to check whether everything we discussed is adequately described. At that time-point additions are still welcome.” |
